# Harnessing Protein Corona for Biomimetic Nanomedicine Design

**DOI:** 10.3390/biomimetics7030126

**Published:** 2022-09-06

**Authors:** Zhidong Chen, Xu Chen, Juyang Huang, Junqing Wang, Zhe Wang

**Affiliations:** 1 Department of Pathology, The Eighth Affiliated Hospital, Sun Yat-sen University, Shenzhen, 518000, China; 2 School of Pharmaceutical Sciences, Shenzhen Campus of Sun Yat-sen University, Shenzhen, 518000, China

**Keywords:** protein corona, nanomedicine, cell membrane, nanocorona, stealth effect, endogenous protein

## Abstract

Nanoparticles (NPs) are usually treated as multifunctional agents combining several therapeutical applications, like imaging and targeting delivery. However, clinical translation is still largely hindered by several factors, and the rapidly formed protein corona on the surface of NPs is one of them. The formation of protein corona is complicated and irreversible in the biological environment, and protein corona will redefine the “biological identity” of NPs, which will alter the following biological events and therapeutic efficacy. Current understanding of protein corona is still limited and incomplete, and in many cases, protein corona has adverse impacts on nanomedicine, for instance, losing targeting ability, activating the immune response, and rapid clearance. Due to the considerable role of protein corona in NPs’ biological fate, harnessing protein corona to achieve some therapeutic effects through various methods like biomimetic approaches is now treated as a promising way to meet the current challenges in nanomedicine such as poor pharmacokinetic properties, off-target effect, and immunogenicity. This review will first introduce the current understanding of protein corona and summarize the investigation process and technologies. Second, the strategies of harnessing protein corona with biomimetic approaches for nanomedicine design are reviewed. Finally, we discuss the challenges and future outlooks of biomimetic approaches to tune protein corona in nanomedicine.

## 1. Introduction

With many excellent properties, nanoparticles (NPs) have been treated as promising drug delivery systems for therapeutic purposes, like efficient and safe drug delivery without off-target effects [1,2]. Even though much attention has been paid to the development of NPs, there is still a huge gap between current NP therapy and efficient delivery systems [2]. One of the most critical factors that hinder the clinical translation of NP therapy is the complicated and unpredictable interaction between NPs and biological fluids that occur after the administration of NPs [3]. The substances in biological fluids, especially proteins, will absorb on the surface of NPs and rapidly form an external layer called protein corona through all kinds of nano-bio interactions and biochemical driving forces [4]. This layer consists of many biomolecules, like albumin, complement protein, and apolipoprotein, which will redefine the “biological identity” of NPs [5]. Not only will the physicochemical properties of NPs change, including size, shape, and surface properties, but also their biological fate and therapeutic effects, such as circulation time, biodistribution, stability, immune system activation, cellular uptake, targeting effect, therapeutic efficacy, and toxicity [6,7,8]. As Yi-Feng Wang et al. indicated, the protein corona will influence the cellular transportation mechanism and intracellular distribution of NPs [9]. They suggested that the interaction of cationic liposomes with cells would switch from energy-independent membrane fusion to energy-dependent endocytosis under the presence of protein corona. They also found that in serum-free conditions (without protein corona), the NPs are mainly distributed in the nucleus, different from the NPs with serum, which is mainly distributed in lysosomes. Therefore, there is an urgent need to explore the rules and mechanisms of protein corona formation and understand the influence of protein corona in NPs therapy, which will provide guidance for the rational design of NPs and accelerate the clinical translation speed of NP therapy.

**Figure 1:**
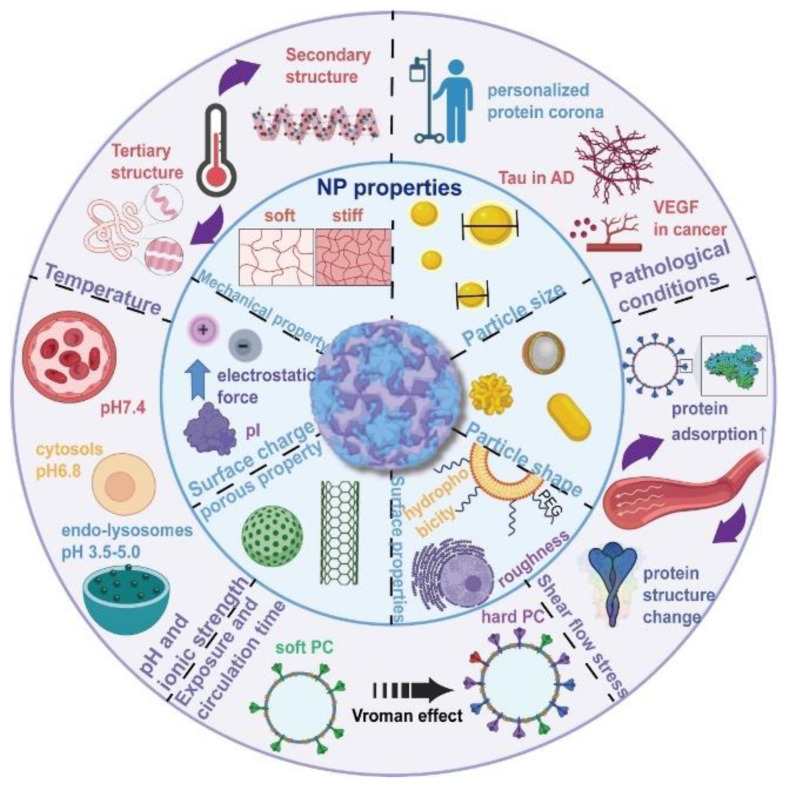
The major factors that influence the formation of protein corona can be divided into two categorized properties: NPs properties and biological environment properties. NPs properties mainly consist of particle size, shape, surface properties, porous properties, surface charge, and mechanical properties. Biological environment properties mainly consist of temperature, pH and ionic strength, exposure and circulation time, shear flow stress, and pathological conditions [19]. Copyright 2022, Elsevier.

The formation of protein corona is rapid, temporally dynamic, contextually dependent, stochastic, and irreversible [2,10,11]. Current studies suggest that protein corona will be established rapidly and then experience a dynamic process in the biological fluids, which means that the composition will change over time [10,12]. In biological fluids, the proteins with low affinity and high content will initially bind to the surface of NPs, and they will be replaced by proteins with high affinity and low content as time goes by [13]. Similarly, the protein corona will also experience a significant change during the process of intracellular delivery. With several advanced analytical techniques, Chunying Chen’s team revealed the dynamic evolution of the protein corona composition during cell transport for the first time [14]. After entering the lysosomes and cytoplasm in the cell, there was a great difference in NPs’ protein corona, considerably influencing cell glycolysis, cell energy metabolism, and the cell lipid metabolism process. There are numerous factors influencing the formation of the protein corona, and these factors can mainly be divided into two categorized properties: NPs properties and biological environment properties [2,11,15,16,17,18,19], which are listed in Figure 1. The source of the biological media (e.g., plasma) and protein origins are also considered significant factors in protein corona formation. Around these factors one of the primary factors is the proteome profile of the biological environment, which is largely influenced by personal factors [20]. Hence, the concept of “personalized protein corona” is widely accepted [19], which means that some personal factors, like disease states, pregnancy, gender, and age, will also have a significant influence on protein corona formation [21,22,23]. Since the role and impact of protein corona in NPs-based therapy cannot be overlooked, exploring and understanding the influencing factors of protein corona formation is a critical step in understanding the biological fate of NPs and a prerequisite for the rational design of NPs-based therapy [24].

It is well known that the protein corona will redefine the “biological identity” of NPs and significantly affect the NPs’ therapeutic effects [19]. However, the current understanding of the relationships between protein corona and the biological fates of NPs is still fragmented and incomplete [2]. In many cases, protein corona is uncontrollable, and it will result in adverse effects on NPs, for example, activating the immune system, triggering complement activation, and opsonization, which will cause the quick removal from the blood through the mononuclear phagocytic system (MPS) [25]. Jianbin Mo et al. found that the protein corona around black phosphorus (BP) nanomaterials will induce immunotoxicity and immune perturbation in macrophages and increase the uptake efficiency of macrophages, largely influencing the circulation time and therapeutic effects of BP therapy [26]. Another adverse influence of protein corona is the disability of targeting ligands on the NPs’ surface. The functionalization of NPs’ surface for targeting ability is a powerful and widely used method for improved therapeutic outcomes in NP therapy, but the targeting ability may disappear when NPs enter the biological environment because of protein corona [27,28]. Anna Salvati et al. showed that the transferrin-conjugated NPs lost their targeting specificity when placed in biological fluids, because the protein corona shields the transferrin on the NPs’ surface from binding to its receptors, resulting in the loss of targeting ability [29]. Yazhen Wang and co-workers also revealed that the protein corona under the cerebrospinal fluid would shield the targeting motif of transferrin-modified NPs, cause the loss of active targeting specificity, and alter the interaction of NPs with cells [30]. Besides influencing the therapeutical effect of NP therapy, the protein corona will also be the mechanism of diseases in some cases. In the research of Zhenzhen Wang and co-workers, they indicated that the specific composition in protein corona could mediate a particular pathogenic process in a clinically relevant disease [31]. Specifically, they found that transforming growth factor β1 (TGF-β1) in the protein corona will subsequently induce the development of lung fibrosis and exacerbate the occurrence of pulmonary fibrosis.

Even though many adverse influences of protein corona on NPs’ therapeutical effects have been discovered and verified, the formation of protein corona is proven to be an indispensable factor for some purposes in some cases [32]. Harnessing protein corona by all kinds of methods becomes a promising way to improve the efficiency and expand the applications of NPs therapy [33]. Recent studies have shown that the developed selective organ targeting (SORT) NPs could achieve targeting delivery to non-liver tissues, and the underlying mechanism was mainly the specific protein corona around the NPs [34,35]. The special components, called SORT molecules in these NPs, could determine which proteins will avidly adsorb to the NPs’ surface. In other words, the SORT molecules will endow the NPs with different protein corona and subsequently manipulate the following biodistribution. Avoiding the formation or controlling protein corona composition also shows the potential to increase the circulation time of NPs and avoid non-specific cellular uptake. Poly (ethylene glycol) (PEG) has been widely used to suppress the formation of protein corona for lower cellular uptake of NPs, and a recent study showed that the effects of PEG not only result from the reduced protein corona formation but also from the change of protein corona composition around NPs, which is essential to increase blood circulation time [32]. The protein corona of the NPs modified with PEG comprises a large number of clusterin proteins, which may be the major reason for the reduced macrophage uptake. The protein corona can also confer the targeting ability of NPs when certain proteins that have a high affinity to specific receptors can be recruited in protein corona, which might be a potential strategy to overcome the shortcomings of the current targeting delivery approaches. This mechanism is one of the factors that contribute to the success of Onpattro (patisiran), which is the first ever lipid nanoparticle-based short interfering RNA drug for the treatment of hereditary amyloidogenic transthyretin (ATTRv) amyloidosis [36,37,38]. These NPs formulations show remarkable affinity to the liver and high hepatocyte accumulation, resulting from a large number of apolipoprotein E (ApoE) being adsorbed into the protein corona. The ApoE in the protein corona can act as natural and effective targeting ligands, which result in the uptake of NPs by endocytosis through ApoE-dependent low-density lipoprotein receptors (LDLR) on the surface of hepatocytes [39]. Another example of controlling protein corona composition for targeting ability is the NPs system modified with transferrin (Tf)-binding peptide, which was rationally designed from several computational methods [40]. This peptide can effectively bind the Tf in the serum and endow the NPs with Tf-abundant protein corona without altering the original Tf biological function. The effect of protein corona modulation by this Tf-binding peptide would confer the brain-targeting ability to NPs system, and this approach was widely used in some following research [41,42,43].

As a novel and potential method, biomimetic nanotechnology has shown its power and promise in drug delivery systems because it has the potential to overcome the obstacles associated with current nanomedicine. Current NPs are mainly synthetic and usually impeded by various physiological barriers and unexpected biological effects because of their exogenous nature [44,45,46]. Natural substances used in biomimetic nanotechnologies, such as viruses and cells, have evolved for some biological effects, which can be utilized in nanomedicine to overcome the disadvantages of synthetic NPs. The biomimetic approach is versatile with unlimited potential, and many strategies have been developed with the biomimetic approach in nanomedicine [7,47,48,49]. The biomimetic approach aims to mimic natural biological mechanisms and transfer specific natural functionalities to synthetic nanoparticles to achieve therapeutic outcomes or avoid the adverse effects of current synthetic nanomedicine [49]. For example, the NPs decorated with cell membranes from different kinds of cells like erythrocytes (red blood cell [RBC]) can superiorly reduce undesired immune responses, evade elimination by macrophages, and bypass systemic clearance [50]. These NPs with RBC cell membranes show long circulation time and gorgeous therapeutic efficacy [51]. In the case of viruses and bacteria, they have evolved to evade the host immune system and enter a target cell, which is also an ideal approach for certain biodistributions or targeting abilities [7]. The biomimetic approach refers to a wide range, including cell membrane decoration [51], virus-like particles (VLPs) [52], exosome vectors [53], endogenous protein coating [54], and natural ligands modification [55], and there are more and more biomimetic strategies under research [44,45,49].

Taking the potential of biomimetic approaches and the non-negligible effects of protein corona in NP therapy into consideration, precisely controlling protein corona with biomimetic approaches is a promising method to achieve some therapeutic purposes, like avoiding the non-specific cellular uptake by macrophages and targeted delivery [56]. This review will mainly focus on the current development of biomimetic approaches used in NP therapy for controlling protein corona around the NPs. The general research processes and investigation technologies for protein corona will be discussed first, and we will next introduce the biomimetic approach for controlling protein corona. The strategy of the biomimetic approach discussed in this review is divided into three parts, including cell membrane decoration, endogenous protein coating, and biomolecule modification. Finally, we will discuss the limitations of current biomimetic approaches for controlling protein corona formation and ponders the challenges ahead in this field.

## 2. Investigation of the Protein Corona

**Figure 2:**
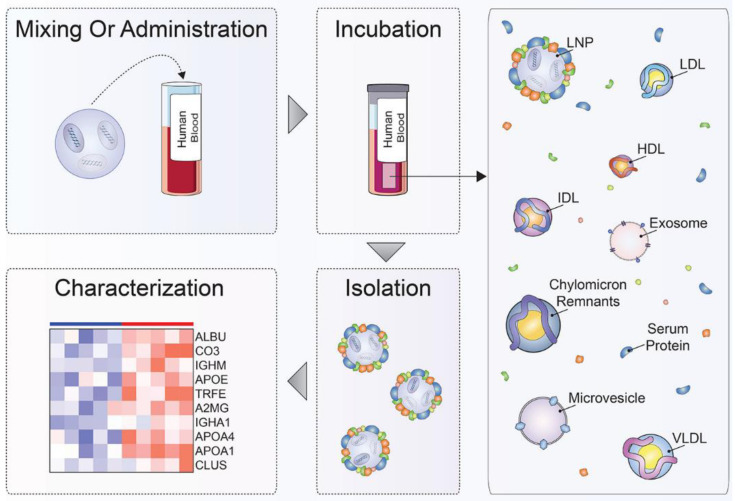
The general research process of the investigation of the protein corona. This process can be divided into four steps, mixing or administration, incubation, isolation, and characterization, and each step has special and important requirements [57]. Copyright 2020, American Chemical Society.

The general research process utilized in the investigation of protein corona is shown in Figure 2 [57]. This process can be generally divided into four steps, mixing or administration, incubation, isolation, and characterization, and each step has unique and stringent requirements. To simulate the real situations that NPs may encounter after administration as closely as possible, the first step of protein corona investigation is choosing the appropriate conditions to form protein corona, including incubation with suitable conditions or administration in animal models or patients. This step might be the most crucial part of the protein corona investigation because it determines the concrete situation of protein corona that will be analyzed in the following steps. This step should consider numerous factors, including but not limited to types of biological fluids, species, intended application, administration route, disease, gender, and incubation temperate [2,57,58]. After obtaining protein corona in relatively correct conditions, the following steps of protein corona investigation involve the isolation, purification, and characterization of the protein corona. The isolation and purification are also critical steps in the protein corona investigation. There are also some critical requirements, such as preserving the original protein corona composition during isolation and separating the NP–protein corona complex from endogenous biomacromolecules like lipoproteins and extracellular vesicles [59,60,61]. The isolation and purification techniques used to isolate the NP–protein corona complex from the surrounding matrices include centrifugation, magnetism, and chromatographic methods. Specifically, the most widely used isolation and purification methods consist of centrifugation, size exclusion chromatography, asymmetric flow-field-flow fractionation, magnetism separation, and cross-linking, which have their own advantages and disadvantages [57,60]. Many factors should be considered when choosing the isolation methods, like the kinds and physicochemical parameters of NPs, the surrounding matrix, possible unintended interactions, and the desired fate of the protein corona [60]. The final step of protein corona investigation is to characterize obtained protein corona with several analytical technologies, like sodium dodecyl sulfate-polyacrylamide gel electrophoresis (SDS-PAGE) and liquid chromatography-tandem mass spectrometry (LC-MS/MS) [62].

**Figure 3:**
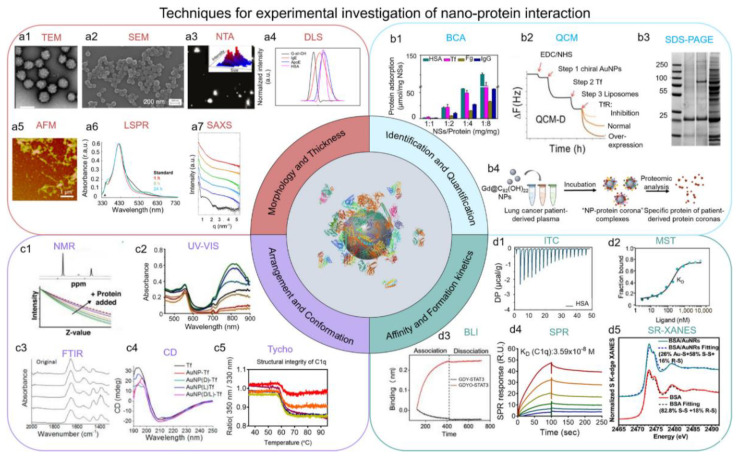
The research technologies used for the investigation of the protein corona are mainly divided into four parts: morphology and thickness; identification and quantification; arrangement and conformation; and affinity and formation kinetics [3]. Copyright 2022, American Chemical Society.

As discussed above, there is still a lack of understanding of several fundamental principles of the behavior of NPs in vivo and their long-term biological effects. One of the crucial reasons is the scarcity of high sensitivity, high resolution in situ analysis methods to investigate the protein corona through time, and the development of advanced methodology has become a bottleneck in the field of NPs and protein corona. Many analytical and biophysical techniques have been developed to investigate protein corona. The scope of these technologies is primarily made up of four parts, including morphology and thickness; identification and quantification; arrangement and conformation; affinity and formation kinetics, which have been discussed in detail in the previous review [3] (Figure 3). The combination of the techniques will be the future direction in this field. One example is the method developed by Chunying Chen’s team [63]. To elucidate the role of NP–protein interactions and the subsequent biological fates of NPs, they proposed a trinity research strategy of in situ characterization, metabolic analysis, proteomics, and molecular simulation to reveal the biodistribution, degradation, metabolism, and biochemical transformation of NPs in vivo through the integration of a variety of advanced analysis techniques and computational methods. Through this advanced method, they elucidate that the biodistribution of NPs after intravenous injection is mainly mediated by the protein corona. To be specific, they revealed that the apolipoproteins (eg, ApoE, ApoJ) in the protein corona mainly mediated the enrichment of NPs in Kupffer cells from the liver and red myeloid macrophages from the spleen, which is similar to the mechanism of the example discussed above [35,36]. Despite much research focusing on the formation mechanisms of the protein corona, the influence factors of the protein corona, and related research techniques, there is still a long way to fully understand protein corona and its biological effects with vigorous and accurate methodological approaches and technologies [64]. The development of protein corona characterization techniques will make a difference in the reproducibility and transparency of NP therapy, minimize misinterpretations to the maximum extent, and therefore accelerate the clinical translation speed of NP therapy [64].

## 3. Biomimetic Approach to Harness Protein Corona

In order to rationally design NP therapies with good safety, biocompatibility, favorable biodistribution, and high efficacy, it is necessary to precisely consider the interaction between NPs and biological fluids and the formation of protein corona [65]. Harnessing protein corona for particular therapeutic purposes is now treated as a promising strategy in nanomedicine because an ideal protein corona can have positive effects like targeting ability [33]. The biomimetic approach is now widely used in nanomedicine, especially in NPs, also called bio-inspired NPs. The bionic components in bio-inspired NPs will largely alter the properties and identity of original NPs, resulting in an entirely different protein corona and thus mediating the biological fates and therapeutic outcomes of NPs [44]. The details of recent studies involving the biomimetic approach in nanomedicine to harness protein corona are presented in Table 1. This section will discuss the current development of bio-inspired NPs with good efficacy by controlling the protein corona, mainly divided into cell membrane decoration, endogenous protein coating, and biomolecule modification.

### 3.1. Cell Membrane Decoration

Cell membrane decoration is one of the most powerful and widely used methods in the biomimetic approach. This decoration can endow NPs with many gorgeous properties by mimicking the natural functionality of various cell types, such as long circulation time and reduced undesired immune responses [99,100]. According to the therapeutic requirements, a variety of cell types have been considered for cell membrane decoration, including but not limited to red blood cells (RBC), white blood cells (WBC), platelets, cancer cells, stem cells, bacterium, and other unconventional cell sources [101]. Herein, we will summarize the decorating approaches via RBC membrane, WBC membrane, platelets, and exosomes since the decoration of these membrane types is more likely to function by controlling and harnessing protein corona.

#### 3.1.1. Red Blood Cell (RBC) Membrane Decoration

Red blood cell (RBC) membrane decoration is a popular and important method in bio-inspired NPs, and the RBC membrane is usually treated as a natural long-circulation delivery vehicle [102]. These biomimetic decorations have been shown to endow NPs with longer circulation time and less uptake rate into MPS organs without unexpected toxicity and accelerated clearance rate [103]. The ability of the RBC membrane is owed to the abundant crucial self-markers, including CD47 proteins, CD59 proteins, peptides, and glycans, whose original functions are protecting RBC from being cleared by the immune system and giving the RBC a long circulation time [99].

Red blood cell (RBC) membrane decoration is a popular and important composition in bio-inspired NPs, and RBC membranes are usually treated as natural long-circulation delivery vehicles [102]. These biomimetic decorations have been shown to endow NPs with longer circulation time and less uptake rate into MPS organs without unexpected toxicity and accelerated clearance rate [103]. The ability of RBC membranes is owed to abundant crucial self-markers, including CD47 proteins, CD59 proteins, peptides, and glycans, whose original function is to protect RBC from being cleared by the immune system and give the RBC a long circulation time [99].

**Figure 4:**
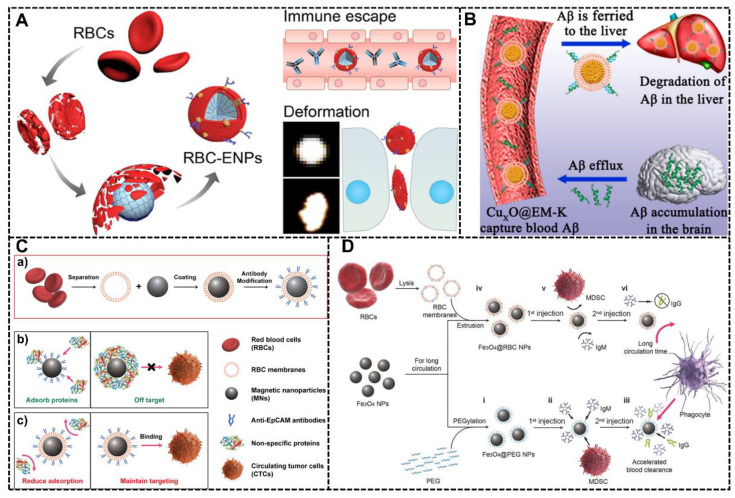
RBC membrane decoration to harness protein corona. (**A**) The RBC-mimic system (RBC-ENPs) was constructed based on the RBC membrane and poly (ethylene glycol) diacrylate (PEGDA) NPs. The RBC cell membrane decoration endowed this system with the ability of immune escape by controlling the composition of the protein corona and deformation for better tumor penetration [50]. Copyright 2022, American Chemical Society. (**B**) A biomimetic system with RBC membrane decoration (CuxO@EM-K). The RBC membrane would impede the formation of the protein corona and minimize immunogenicity, facilitating the ability to adsorb Aβ efficiently for a much longer time [68]. Copyright 2020, American Chemical Society. (**C**) The preparation and mechanism of RBC-IMNs system for enhanced isolation of CTCs. (a) The RBC membrane was decorated onto the IMSs before antibody modification; (b) The IMNs without the RBC decoration will absorb proteins in biological fluids, and the protein corona will largely influence the isolation efficiency and cause the off-target effect; (c) The IMNs with RBC membrane decoration will reduce the formation of the protein corona, and therefore maintain the targeting ability of IMNs for CTCs isolation in biological fluids [67]. Copyright 2019, American Chemical Society. (**D**) The RBC membrane decoration is an ideal superior alternative to PEG for prolonged circulation time and escapes from clearance by the immune system [66]. Copyright 2015 Wiley-VCH.

Yunqiu Miao et al. developed a novel NPs system whose surface was decorated with natural RBC membranes [50]. This RBC-mimic system was constructed based on RBC membranes and poly (ethylene glycol) diacrylate (PEGDA) hydrogel nanoparticles, and due to the RBC membrane, this system exhibited many gorgeous properties, including evading the immune system efficiently, penetrating narrow tissue extracellular space of tumors and accumulating in diseased tissues (Figure 4A). The protein corona of this system in vivo was investigated with liquid chromatography-tandem mass spectrometry (LC-MS/MS), and the results showed that this RBC-mimic system has the lowest immunoglobulin adsorption in protein corona, which may be the primary reason for less immunogenicity, reduced opsonization in macrophages and ultralong circulation time. Through in vivo tumor penetration and an in vivo anti-tumor efficacy test, the efficacy of this RBC-mimic system was certified.

Mengmeng Ma et al. also designed an intriguing bio-inspired NPs system with RBC membrane decoration, which is used for clearance of peripheral Aβ associated with AD [68]. Recent research shows that the clearance of peripheral Aβ may be a promising way to overcome the BBB obstacle for halting the progression of AD, but the effect is largely influenced by the formation of protein corona and the activation of immune responses. Therefore, they tried to introduce the RBC membrane to overcome the problems that came from protein corona. The RBC membrane in this system plays a considerably significant role: on the one hand, this decoration can prevent the formation of the protein corona, which is necessary for the maintenance of Aβ-targeting capability to clear the Aβ associated with AD; on the other hand, this decoration can minimize immunogenicity by the resistance of protein corona formation, which is crucial for the effective absorbing Aβ (Figure 4B). Through in vivo study, this system was proven to not only reduce the Aβ burden in the blood and brain but also reverse learning and memory impairments.

Another NPs system with RBC membrane decoration to harness protein corona was fabricated by Qian-Fang Meng and co-workers [67]. The RBC decorated NPs system was immunomagnetic micro/nanoparticles (IMNs), which have been frequently used to enrich the rare circulating tumor cells (CTCs) from clinical blood samples for early diagnosis of cancers and post-therapy evaluation. The greatest challenge the IMNs face in isolating CTCs is the formation of protein corona in biological fluids because protein corona largely hinders the interaction between IMNs and CTCs and decreases the targeting ability of IMNs. Therefore, in this study, they tried to prevent the formation of protein corona and preserve the targeting ability of IMNs by the RBC membrane decoration (Figure 4C). By analyzing the protein corona formation in IMNs and RBC-IMNs along with CTC-isolation performance between commercial Dynabeads and RBC-IMNs, they demonstrated that the RBC-IMNs would not form protein corona and maintain targeting ability in the biological environment, showing their excellent potential in clinical translation and application.

The study of Lang Rao et al. demonstrated that the RBC membrane decoration is an ideal superior alternative to PEG [66]. The RBC membrane will not only prevent the formation of protein corona around the NPs as PEG but also help NPs to escape immune clearance and improve the circulation time through the signal regulatory protein-alpha (SIRP-α) of the CD47, the “do not eat me” marker on the RBC surface (Figure 4D). Compared with PEG decoration, the system with RBC membrane decoration shows a longer circulation time with pharmacokinetic studies in vivo. The in vivo toxicity of this NP system with RBC membrane decoration was studied by blood biochemistry, hematology testing, and histology analysis, showing the gorgeous potential of this NP system.

#### 3.1.2. White Blood Cell (WBC) Membrane Decoration

**Figure 5:**
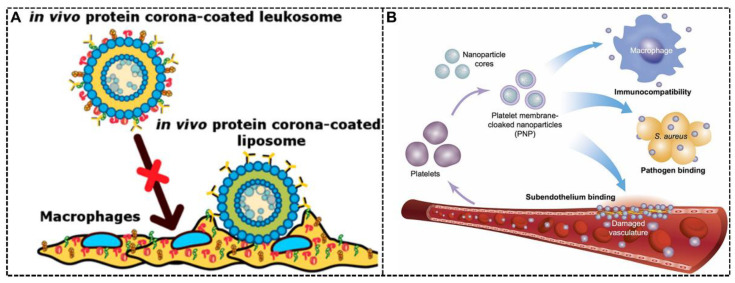
WBC and platelet membrane decoration to harness protein corona. (**A**) Based on the WBC membranes’ decoration, the biomimetic liposomes called leukosomes showed reduced nonspecific interactions and more adsorption of specific proteins over others, resulting in the ability to target inflamed endothelium and avoid clearance by macrophages [73]. Copyright 2017, American Chemical Society. (**B**) Poly(lactic-co-glycolic acid) (PLGA) NPs were decorated with the platelet membrane, showing reduced cellular uptake by macrophage cells and better therapeutic effects [75]. Copyright 2015, Springer Nature.

The white blood cell (WBC) membrane is another cell membrane showing excellent potential in bio-inspired NPs. [104] Numerous advantages of the RBC membrane have been discussed above. Nevertheless, it lacks the targeting property, indicating the need for conjugating ligands if targeting ability is crucial and needed in precision medicine [99]. The WBC shows inherent homing properties to inflammation or other diseased regions, which can cover the deficiency of RBC in bio-inspired NPs [101]. According to the granularity and morphology, WBC can be divided into five major types: neutrophils, monocytes/macrophages, eosinophils, basophils, and lymphocytes [99]. The inherent homing property of WBC has been taken advantage of in bio-inspired NPs, for example, macrophages (RAW 264.7) and their tumor/inflammation homing ability [105]. Apart from a homing property, WBC membranes also show the ability to control protein corona for intriguing therapeutic effects. Claudia Corbo et al. fabricated biomimetic liposomes with WBC membranes called leukosomes, and this system showed a gorgeous ability to target inflamed endothelium and avoid clearance by the immune system [73]. This system showed longer circulation time and improved accumulation in inflamed tissues in vitro and in vivo compared with the control group. Through a time-dependent quantitative and qualitative analysis of protein corona around this bio-inspired NP system, the mechanism of this system was revealed. The WBC membranes would affect the composition and amounts of protein corona, not only hindering the nonspecific interactions through the masking effect but also facilitating the adsorption of specific proteins and endowing the NPs with targeting ability (Figure 5A).

#### 3.1.3. Platelet Membrane Decoration

Platelet originated from megakaryocyte progenitor cells and is also a widely explored cell type for cell membrane decoration [100]. Similar to RBC, many self-markers exist in the platelet membrane, which indicates that the platelet membranes can also be used to evade complement-mediated immune activation for a long circulation time [99]. It is certified that the platelet can quickly respond to vascular damage, have tumor-targeting capabilities [106], prolong blood circulation, and reduce hepatic uptake, making the platelet membrane an intriguing biomimetic approach in NP therapy [107]. It is well known that the primary function of platelet is hemostasis by inducing platelet aggregation with the presence of Gp (glycoprotein) IIb/IIIa receptors, which makes it easy to coat the platelet membrane onto all kinds of NPs and therefore achieve some therapeutic effects [99,108]. Che-Ming J. Hu et al. fabricated a novel NP system decorated with a platelet membrane, showing platelet-mimicking properties like enhanced binding to platelet-adhering pathogens [75]. The authors found that compared with non-decorated NPs, this decoration reduced cellular uptake by macrophage cells and did not induce complement-mediated immune activation, which might contribute to the influence on protein corona formation (Figure 5B). By experimental rat model of coronary restenosis and systemic bacterial infection, this platelet-mimetic NP system with docetaxel and vancomycin was proven to have better therapeutic efficacy.

#### 3.1.4. Exosomes-Based Decoration

**Figure 6:**
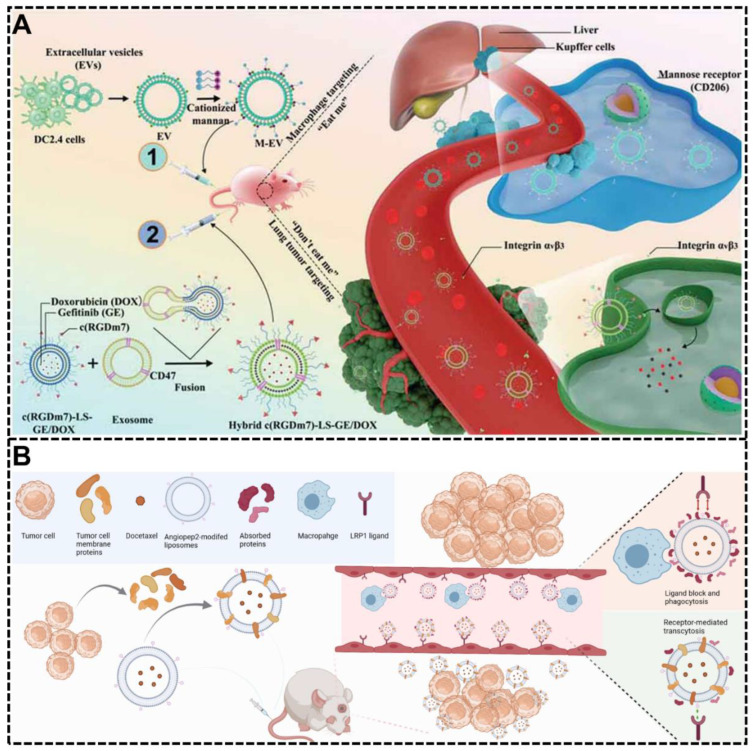
Exosomes-based decoration to harness protein corona. (**A**) A combined “eat me/don’t eat me” strategy with a bio-inspired NP system using exosome-based decoration was proven to have prolonged circulation time and increased tumor accumulation by controlling the protein corona and achieving macrophage escape [76]. Copyright 2020 Wiley-VCH. (**B**) A novel bio-inspired NP system by multifunctional exosome-mimetics (EM) was developed to target glioblastoma (GBM) drug delivery by controlling protein corona formation, escaping phagocytosis, enhancing BBB penetration, and GBM targeting [77]. Copyright 2021, Springer Nature.

Recently, numerous studies have shown that exosomes hold promise as a new generation of biomimetic drug delivery systems due to their unique endogeneity and bioactivity [53]. The exosomes contain many endogenous substances from all kinds of cells, making them an ideal resource for developing bio-inspired NP systems [53,109]. Many bio-inspired NP systems are decorated with exosomes for intriguing therapeutic effects by controlling protein corona formation and composition. Zakia Belhadj et al. developed a combined “eat me/do not eat me” strategy with a bio-inspired NP system by exosome-based decoration [76]. The CD47 was introduced into this NP system through the decoration of CD47-expressing exosomes originating from human serum, which can influence the formation of protein corona and show a strong ability to evade phagocytosis by macrophages through the “do not eat me” signal of CD47 in the surface (Figure 6A). Through the in vitro and in vivo tests, this system showed prolonged circulation time, increased tumor accumulation, and enhanced therapeutic efficacy with fewer negative influences on liver or spleen function. Another bio-inspired NP system used to harness protein corona by exosome-based decoration was developed by Jun-Yong Wu et al. [77]. In their research, multifunctional exosome-mimetics (EM) were developed and decorated with angiopep-2 (Ang) for enhancing glioblastoma (GBM) drug delivery by controlling protein corona (Figure 6B). The exosome-based decoration makes the surface of this NP system with lots of chimeric proteins, which could decrease the formation of the protein corona, escape the phagocytosis by macrophages and retain its natural properties. This system showed enhanced GBM targeting ability and excellent therapeutic effect because of the combination of exosome-based decoration and Ang modification.

### 3.2. Endogenous Protein Coating

Besides cell membrane decoration, endogenous protein coating is another powerful approach in bio-inspired NP therapy. Since the surface properties of NPs are one of the major factors that determine the constitution of protein corona and the most abundant component of protein corona is protein, the endogenous protein coating has also been extensively studied to control the formation of functional protein corona because the pre-coated endogenous protein can act as a biomimetic component on the surface of NPs for some special therapeutic effects by affecting the protein corona [85]. Based on therapeutic effects, the purpose of endogenous protein coating to harness protein corona can be mainly divided into a stealth effect for prolonged circulation and lower immune activation and targeting ability.

#### 3.2.1. Endogenous Protein Coating for Stealth Effect

**Figure 7:**
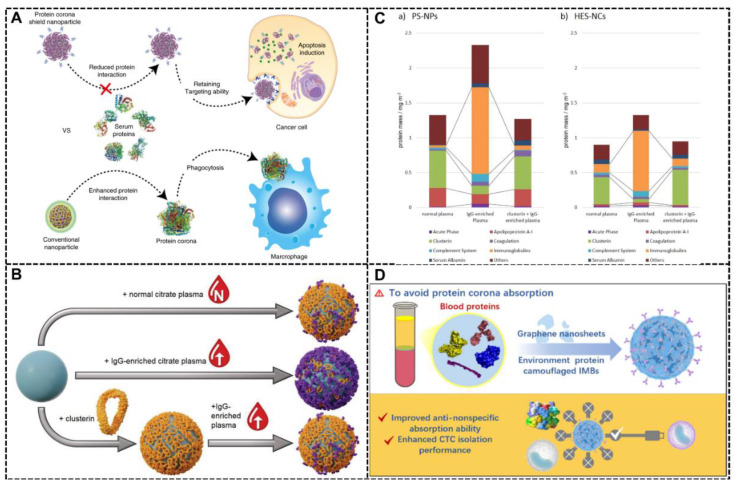
Endogenous protein coating for stealth effect. (**A**) The system with supramolecular precoating shows the effect of protein corona shield by reducing the interaction between the NPs and biological fluids and preventing the protein corona formation for retaining targeting ability [54]. Copyright 2018, Springer Nature. (**B**) The NP system with stealth protein clusterin pre-coated (bottom) showed reduced cellular internalization despite being incubated in the artificially IgG-enriched citrate plasma [84]. Copyright 2019 Wiley-VCH. (**C**) The composition of the protein corona in two different NP systems called (a) PS-NPs and (b) HES-NCs under several conditions, including incubation with normal plasma, IgG-enriched plasma, or IgG-enriched plasma after preincubation with clusterin. This result was analyzed via LC-MS detected by a Pierce 660 nm protein assay [84]. Copyright 2019 Wiley-VCH. (**D**) The HSA precoating avoids the protein corona absorption and formation, which is beneficial for improved anti-nonspecific absorption ability and enhanced CTC isolation performance [88]. Copyright 2022, American Chemical Society.

The strategy of endogenous protein coating is widely used for stealth effect, which means regulating the NP-biological fluids interactions, preventing the NPs from clearance by macrophages, and endowing the NPs with longer circulation time [78]. Jun Yong Oh et al. presented an intriguing NP system with an endogenous protein coating strategy, endowing this system with the stealth effect, which means maintaining the targeting ability of the targeting ligands and prolonging the circulation time in blood by enabling escape from MPS clearance [54]. In this NP system, the HER2-binding affibody worked as a targeting ligand, and the biomimetic part, which consists of glutathione-S-transferase, was pre-coated around this NP system for stealth effect (Figure 7A). The absorbed protein will minimize the interaction between the NPs with biological fluids, prevent protein corona formation, and prevent the internalization by macrophages. With confocal microscopy imaging and cell viability analysis, the stealth effect of the recombinant fusion protein in this system was proven, showing that this NP system was able to evade clearance by macrophages, which is crucial for long circulation time and excellent therapeutic effects in nanomedicine. The targeting ability and therapeutic efficacy were also certified with cell experiments and in vivo tumor model.

Another example of endogenous protein coating for the stealth effect by controlling protein corona formation was shown by Domenik Prozeller et al. They used the stealth protein clusterin (also called apolipoprotein J (ApoJ)) to coat the NPs, successfully preventing the dominant IgG-adsorption and additionally reducing cellular internalization (Figure 7B) [84]. The cell experiments showed that the pre-coating of clusterin resulted in reduced unspecific cell uptake in vitro, which came from its ability to change the formation and composition of the protein corona. Figure 7C presents the composition of the protein corona in two different NP systems called (a) PS-NPs and (b) HES-NCs under several conditions, including incubation with normal plasma, IgG-enriched plasma, or IgG-enriched plasma after preincubation with clusterin. As shown in Figure 7C, compared with the group of “IgG-enriched plasma,” there was a remarkable reduction in the amount of immunoglobulins protein in the composition of the protein corona in the group of “IgG-enriched plasma after preincubation with clusterin,” which might be the reason for the stealth effect in this NP system.

Like RBC membrane decoration, the endogenous protein coating strategy can also benefit the isolation of circulating tumor cell (CTC) enrichment and downstream analysis. Albeit being treated as a promising CTC-isolation platform, the performance of immunomagnetic beads (IMBs) will be destroyed in the biological environment because protein corona may shield the targeting motif and reduce its effectiveness. Endogenous protein coating for preventing protein corona formation is an intriguing method. By comparing the four most abundant serum proteins, including human serum albumin (HSA), fibrinogen, immunoglobulin, and transferrin, Xiaoxi Zhou and co-workers found that the HSA coating endows the IMBs with the highest specificity (CTC isolation performance), anti-nonspecific absorption ability, anti-leukocyte absorption ability and excellent sensitivity (Figure 7D) [88].

In addition to single endogenous protein coating, coating a complex of endogenous protein can also be used to harness protein corona. Francesca Giulimondi et al. fabricated a liposomes system pre-coated with an artificial corona made of human plasma proteins (a complex of endogenous proteins). They found that the protein corona influenced by the artificial corona in the biological environment will control the interaction between liposomes with immune cells. Pre-coating was a novel strategy for escaping sequestration by immune cells and endowing liposomes with prolonged circulation time in vivo [78].

#### 3.2.2. Endogenous Protein Coating for Targeting Effect

Endogenous protein coating can also be used for targeting ability. For example, transferrin (Tf) is treated as a targeting ligand for tumor, or brain targeting due to the overexpress of the transferrin receptor (TfR) in cancer cells and the blood-brain barrier (BBB) endothelial cells [110,111]. Previous studies have shown the adverse effects of protein corona on targeting ligands in NP systems [28,29], and another study found that different types of protein corona have different influences and effects on the targeting ability of NP systems [112]. Therefore, by understanding the mechanism of the influences of protein corona and precisely harnessing protein corona with several methods like endogenous protein coating, it is promising to endow the NPs with gorgeous targeting ability.

**Figure 8:**
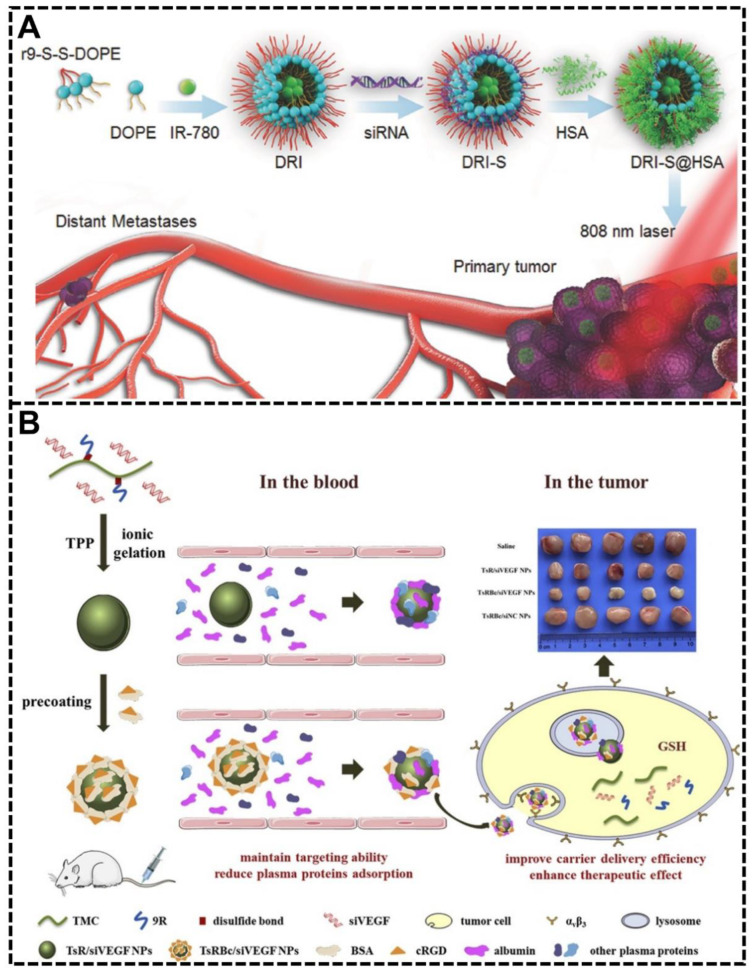
Endogenous protein coating for targeting effect. (**A**) The design and schematic illustration of the NP system (DRI-S@HSA) with endogenous protein albumin coating for long blood circulation time and effective tumor-targeting capability [81]. Copyright 2017, Wiley-VCH. (**B**) A bio-inspired NP system with cyclic RGDyK peptide (cRGD) modified bovine serum albumin (BSA) precoating showed enhanced targeting ability to cancer cells and reduced serum proteins adsorption by reducing the protein corona formation [79]. Copyright 2021, Elsevier.

Haiqiang Cao et al. developed a bio-inspired NP system called albumin biomimetic nanocorona (DRI-S@HSA) [81]. Previous studies have certified the long circulation time of albumin and the increased accumulation rate in tumors on account of the increased need for albumin to obtain amino acids and energy [81]. Therefore, by exploiting the biomimetic functions of serum albumin in this study, this bio-inspired NP system would possess prolonged blood circulation time, effective tumor-targeting capability, high accumulation rate, and deep tumor penetration capability are crucial properties in cancer therapy (Figure 8A). This system showed intriguing results in vivo: the biomimetic albumin coating brought a 2.5 times improvement in tumor accumulation and considerably improved deep penetration ability in tumors compared with the non-coating group. These bio-inspired NPs significantly inhibited tumor growth and prevented lung metastasis of breast cancer.

Han Yang et al. showed another bio-inspired NP system pre-coated with cyclic RGDyK peptide (cRGD) modified bovine serum albumin (BSA) for impeding the formation of the protein corona, enhancing targeting ability to tumor cells, increasing delivery efficiency of nucleic acid drugs and improving therapeutic effects [79]. By preventing the protein corona formation, the pre-coating in this system played a dual role: enhancing the targeting ability of cancer cells and reducing serum protein adsorption for a prolonged circulation time (Figure 8B). Apart from the ability to harness protein corona and tumor targeting, the bio-inspired pre-coating could also increase the stability of this NP system under the lysosomal acid environment and reduce its cytotoxicity. The anti-cancer cell proliferation and anti-tumor proliferation effects were studied in vitro and in vivo, proving that pre-coating the functional biomimetic albumin is an excellent and promising strategy for better drug delivery efficiency and therapeutic effects.

Other endogenous proteins are used for harnessing protein corona, for example, the ApoE protein. The role of ApoE in protein corona has been explored in the recent clinical approved lipid nanoparticle-based short interfering RNA drug Onpattro, and there are also other studies focusing on the role of ApoE coating. In the study of Xiang Lu et al., assisted by the combination of experimental and computational methods, they found that ApoE pre-coating on NP systems will change their pharmacokinetic characteristics and prolong the circulation time without increasing cytotoxicity, which could be mainly attributed to their influence on the formation and composition of protein corona [80].

### 3.3. Biomolecules Modification

Despite being paid much attention to, the active targeting approach still suffers from numerous obstructive factors, like the disability of the targeting effects. Few NPs with active targeting developed to the clinical translation stage, which might be attributed to the exogenous property or the rapid and complicated formation of protein corona [27,113]. In some cases, the biological effects and functions of the introduced active targeting substances in NP system will be impeded by protein corona, and the protein corona under the presence of exogenous substances can even lead to the increased clearance of NPs by MPS because of the activated immune system [54]. Apart from many inert (or unknown functions) plasma proteins, many effective constituents can be utilized for several therapeutic effects, like targeting delivery if NPs can recruit and retain specific proteins in the biological fluids [55]. Based on this, an elegant and promising alternative to traditional targeting methods is modifying the formation or composition of protein corona by biomolecule modification without affecting the functions of natural protein corona components. This strategy will endow the NP system with selectivity and targeting ability from biological fluids, which is another biomimetic approach to harness protein corona [40]. Compared with the traditional active targeting strategy, the biomolecule modification to harness protein corona has numerous advantages, including but not limited to stability, less influence from protein corona, low immune responses, and inflammatory reactions [43]. There are many kinds of biomolecules used in this strategy, and in this section, we will mainly discuss biomimetic peptide modification and other biomolecule modifications.

#### 3.3.1. Biomimetic Peptides Modification

Biomimetic peptides, which means the peptides designed from natural protein in some biological process for special therapeutic effects [114], have been widely used in NPs modification to harness the protein corona. Zui Zhang et al. developed a novel bio-inspired NP system with a short nontoxic peptide derived from Aβ_1-42_ for brain-targeted delivery by controlling the protein corona component [55]. The biomimetic peptides (here called SP) were derived from Aβ_1-42_ and devoid of neurotoxicity. As trans-BBB efflux of Aβ into peripheral blood circulation is the significant pathway of physiological clearance with ApoE, ApoJ, and ApoA1 as chaperones, the Aβ and its derived peptides like SP could interact with the lipid-binding domain of the brain-targeting apolipoproteins (i.e., ApoE, ApoJ, and ApoA1) when placed on the NPs’ surface, therefore precisely modulating the composition and functions of the protein corona. Due to the SP, the abundant brain targeting apolipoproteins in the protein corona had the correct direction for multiple receptors recognition and therefore, facilitating the brain transport via LRP1/LRP2/SR-B1 mediated transcytosis. Through many experiments, the concept discussed above was certified, and this NP system was proven to have excellent brain targeting ability and intriguing anti-cancer effect without the problem of immune compatibility.

**Figure 9:**
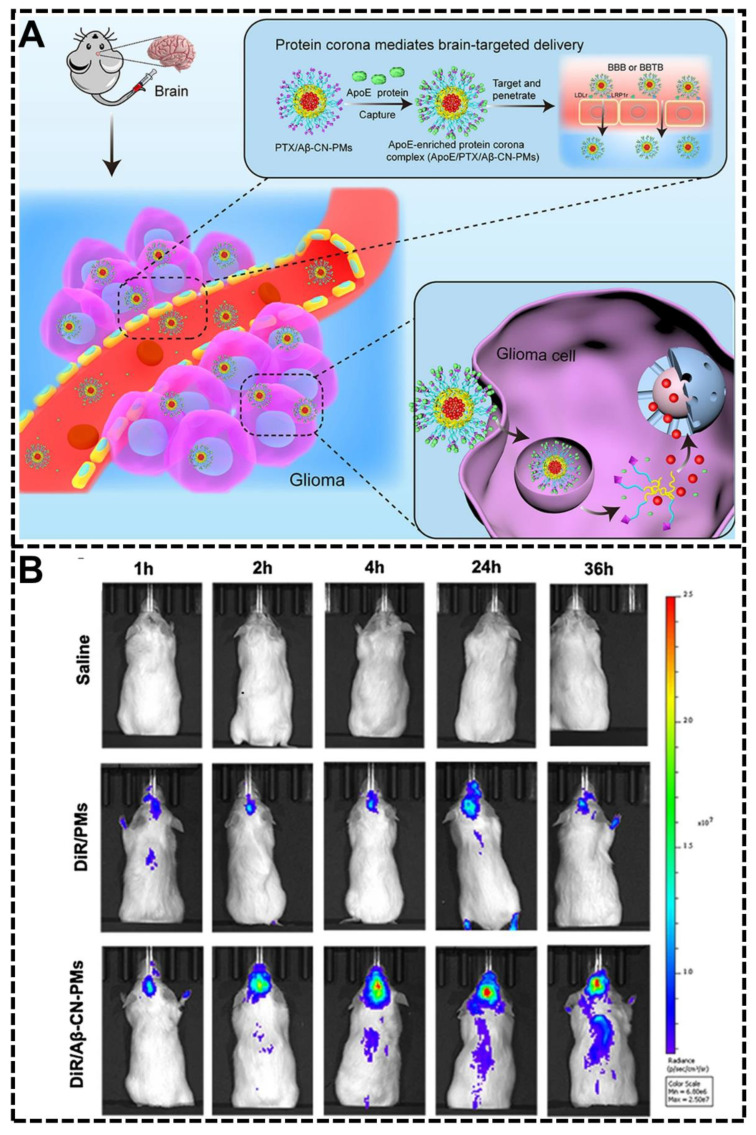
Biomimetic peptides modification to harness protein corona. (**A**) The Aβ-CN peptide modification will capture the ApoE in the biological fluids and form the ApoE-enriched protein corona for brain-targeting [92]. Copyright 2021, Springer Nature. (**B**) The in vivo fluorescence imaging of orthotopic glioma-bearing mice treated with saline, DiR/PMs, and DiR/Aβ-CN-PMs at different time points [92]. Copyright 2021, Springer Nature.

Zhe-Ao Zhang et al. also designed a biomimetic peptide-modified NP system for brain target delivery by controlling the composition of protein corona [92]. The biomimetic peptide used in this study was the 11-amino acid fragment amyloid β-protein (Aβ)_25–35_ (called Aβ-CN peptide), a widely used substitute for the full-length peptide Aβ_1–42_. Similar to the example discussed above, this bio-inspired NP system with the modification of the Aβ-CN peptide could also form an ApoE-enriched protein corona. The receptor-binding domain of the ApoE would bind the low-density lipoprotein receptor (LDLr) and LDLr-related protein one receptor (LRP1r) with high affinity in the blood-brain barrier and glioma and therefore facilitate the brain-targeted delivery (Figure 9A). The in vivo fluorescence imaging of orthotopic glioma-bearing mice treated with saline, DiR/PMs, and DiR/Aβ-CN-PMs at 1 h, 2 h, 4 h, 24 h, and 36 h was exhibited in Figure 9B. The DiR/Aβ-CN-PMs group showed the strongest fluorescence intensity in the brain at any given time point, showing the good brain-targeting ability of this NP system. Through various experiments, including the protein corona characterization, in vitro and in vivo experiments, the tumor-targeting efficacy and anti-glioma effects were investigated in this research, showing the excellent potential of the brain-targeting ability by harnessing the protein corona.

Another example of biomimetic peptides for controlling the protein corona composition in NPs therapy is a peptide called CDX, which is derived from the loop II region of the snake neurotoxin candoxin and shows potential in brain-targeted drug delivery on account of its high binding affinity with the nicotine acetylcholine receptors (nAChRs) [115]. The nAChRs are highly expressed on the capillary endothelium of the brain, and its ligands, like snake neurotoxin candoxin and its biomimetic peptide CDX, were believed to endow NP systems with peptide-based transvascular delivery to the brain. Based on CDX, another peptide called ^D^CDX was developed, which is the retro-inverso peptide analog of CDX. ^D^CDX peptide shows better stability in the biological environment (like the serum), significantly higher transcytosis efficiency in the blood-brain barrier monolayer, and stronger targeting efficiency than CDX peptide [116]. Nevertheless, the NP systems modified with ^D^CDX were proven immunogenic, making it an inappropriate modification ligand in NPs therapy. In the study of Juan Guan and co-workers, they tried to understand the rule and mechanism of the interaction between biological fluids and ^D^CDX functionalized NP systems, and they found that the low immune compatibility of ^D^CDX-modified NPs might come from the enhanced absorption of IgM in the protein corona [89]. The increased IgM in protein corona will bring about many adverse effects, like rapid clearance by MPS and enhanced immunogenicity, and therefore, it is necessary to design a peptide maintaining brain-targeting ability with good immune compatibility. By the computer-aided means, Rosetta peptide dock program in this research, they successfully developed a peptide called D8 based on ^D^CDX for modification in brain-targeting NP systems with improved immune compatibility and reserved brain-targeting ability. The design rule of D8 is modulating the composition of protein corona and reducing its affinity with IgM.

In the research of Mohamadreza Amin et al., they designed a novel NP system with a biomimetic peptide called TAT peptide, which was derived from the transactivator of transcription (TAT) [117]. This peptide has a lot of advantages, including the simplicity of sequence, low cost of preparation and conjugation, and activity against kinds of cancer cells, which enable it to attract lots of attention in NP therapy. In their research, they found that when installed with 100 TAT peptides per NP, the endosomal escape will not be promoted, and the clearance of the TAT peptide containing NPs will be effectively reduced, which results from the protein corona on the TAT peptide containing NPs. The protein corona will not influence the targeting ability of TAT peptides and pharmacokinetics or biodistribution of NPs. On the contrary, the protein corona in the TAT peptide-containing NPs will shield the active moieties, effectively reduce the clearance of the TAT peptide-containing nanoparticles, and balance pharmacokinetics and tumor penetration through interference with avidity.

In addition to the peptide discussed above, there are still many biomimetic peptides developed for special therapeutic effects by harnessing protein corona, like angiopep-2 (Ang) [77] and cyclic RGDyK peptide (cRGD) [79], which have been discussed above.

#### 3.3.2. Other Biomolecules Modification

Besides peptides, many other biomolecules have been used to modify the NP system to control the protein corona conformation and composition, including but not limited to polysaccharides, cholesterol, and phospholipids.

**Figure 10:**
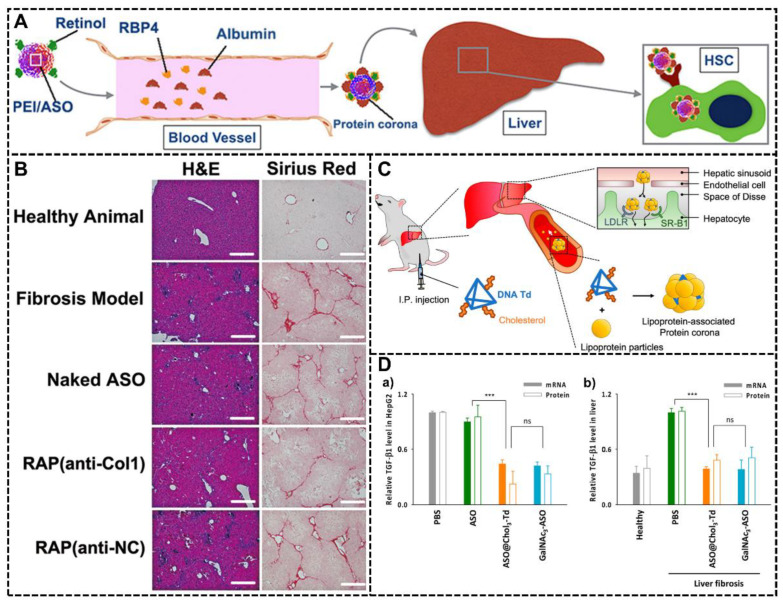
Other biomolecules modification to harness protein corona. (**A**) The schematic diagram of the NP system with retinol molecule modification. The retinol can selectively recruit the RBP4 and form a functional protein corona with numerous RBP4, directing the NPs-PC complex to the HSC in the liver and endowing this NP system with excellent drug delivery efficiency and therapeutic effects [91]. Copyright 2015, American Chemical Society. (**B**) The representative of H&E and Sirius red staining of liver tissue sections. The blue areas indicate the proliferating HSCs, and the red areas indicate the collagen deposition in the fibrotic liver tissues [91]. Copyright 2015, American Chemical Society. (**C**) The NP system with trivalent cholesterol conjugation (Chol3-Td) for ASO in vivo hepatocyte delivery. The cholesterol modification will enhance interaction with lipoproteins in serum and promote the formation of the lipoprotein-associated protein corona. This functional protein corona will facilitate the targeting of ASO delivery to the liver through the interaction with lipoprotein and related receptors like SR-B1 and LDLRs [95]. Copyright 2022, American Chemical Society. (**D**) Comparison of ASO@Chol3-Td with GalNAc3-ASO through estimating the potency of ASO (a) in HepG2 cells in vitro and (b) in liver fibrosis mice in vivo. ***, P < 0.001; ns, nonsignificant [95]. Copyright 2022, American Chemical Society.

In the study of Zhengping Zhang et al., they fabricated a novel NP system with retinol modification [91]. The retinol molecules have a high binding affinity with retinol-binding protein 4 (RBP4), and the complex of retinol and RBP4 can play a significant role in directing the NPs to hepatic stellate cells (HSC), which is considerable in the progression of hepatic fibrosis. In this NP system, the retinol molecules on the surface could facilitate the formation of protein corona composed of relative abundant RBP and therefore showed high targeting ability to HSC and exhibited remarkable therapeutic performances (Figure 10A). This modification could also change the hydrophilicity of the NPs, preventing them from clearance by the immune system. With a CCl_4_-induced murine liver fibrosis model, this NP system with an antisense oligonucleotide (ASO) could effectively direct the NPs into HSC in the liver with excellent targeting ability and therefore suppress the ameliorated hepatic fibrosis for satisfactory therapeutic effects by inhibiting the expression of collagen I. The representative of H&E and Sirius red staining of liver tissue sections was shown in Figure 10B. The blue areas indicated the proliferating HSCs, and the red areas indicated the collagen deposition in the fibrotic liver tissues. The ASO (anti-Col1) was designed to inhibit the expression of collagen I, and the “anti-NC” represented the control group without the inhibition ability of “anti-Col1”. This finding showed a significant improvement in this NP system with the antisense oligonucleotide (ASO) compared with the naked ASO group.

Another study utilizing biomolecule modification to harness protein corona was conducted by Kyoung-Ran Kim and co-workers. They fabricated a DNA tetrahedron platform with trivalent cholesterol conjugation (Chol3-Td) [95]. Cholesterol is of significance in biology, especially in the process of lipid metabolism and lipoprotein delivery. The introduction of cholesterol will vastly increase the interaction between the NP system and lipoproteins in serum, forming the protein corona with abundant lipoproteins that will facilitate the targeting delivery to the liver because many of the liver cells highly express related lipoprotein receptors, like scavenger receptor class B type 1 (SR-B1) and low-density lipoprotein receptors (LDLRs) (Figure 10C). The concept of harnessing the protein corona was proven by proteomic analysis of the protein corona absorbed in this NP system, and one of the most abundant compositions in the protein corona was lipoproteins. To evaluate the therapeutic potential of this NP system, they tried to deliver ASO targeting TGF-β1 mRNA with Chol3-Td for treating liver fibrosis in the mouse model and compared the potency of ASO@Chol3-Td with the clinically approved liver-targeting ligand, trivalent N-acetylgalactosamine (GalNAc3). The result showed that this system was a promising drug delivery system because it could effectively deliver ASO to the liver, downregulate the expression of TGF-β1 mRNA and protein in the liver for the alleviation of liver fibrosis damage, and showed a similar target-gene silencing effect in vitro and in vivo compared with clinically approved liver-targeting ligand GalNAc3 (Figure 10D).

Bo Huang et al. also designed a biomolecules modification NPs system with amphoteric natural starch-stabilized core-shell, which could be treated as a powerful alternative to typical anti-fouling materials such as PEG and zwitterionic polymers for steal effect [97]. The starch modification formed tightly entangled outermost shells combined with strong hydrophilic properties and steric repulsion, which could prevent the formation of protein corona around the NPs and, therefore, enable the NP system to have a longer circulation time in the biological environment. In this study, they also certified that this system could be further functionalized by targeting ligands such as antibodies for additional targeting ability and cell internalization capabilities without influencing protein corona formation in vivo.

## 4. Concluding Remarks and Future Outlook

The properties of NPs and biological milieu factors will determine the formation and composition of the protein corona, and protein corona endows NPs with their new “biological identity”, largely influencing their behaviors in the biological environment and therapeutic effects. Since the considerable roles of the protein corona, harnessing the protein corona with biomimetic approaches is intriguing in nanomedicine as a way to overcome the current obstacles from design to clinical translation, such as pool biocompatibility, off-target effect, toxicity, immunogenicity, and instability. For instance, several examples discussed above try to form an ideal protein corona and endow NPs with the stealth effect for long circulation time and low immunogenicity with biomimetic approaches like cell membrane decoration. However, this concept still has a long way to go to achieve practical nanomedicine for clinical translation.

The first problem in developing NP systems to harness the protein corona is the comprehensive understanding of the formation mechanism, dynamic characteristics, composition, and biological effects of the protein corona. All are fundamental factors in controlling protein corona for practical therapeutic effects in nanomedicine [24]. The gap between the actual protein corona and our understanding mainly comes from the high complexity and heterogeneity of protein corona and the pool technologies we can utilize. Numerous factors will have a significant influence on protein corona, including but not limited to experimental conditions, species, gender, pathobiology, and individual variation [118]. However, the investigation tools are still too rough to fully consider these factors, which makes it difficult to characterize, understand, and utilize the protein corona. For example, in the research of Wei Xiao et al., the in vitro and in vivo protein corona of the same NPs had different impacts on receptor targeting, lysosomal escape, and BBB transcytosis, which means that it is inappropriate to study protein corona in vitro alone in some research [28]. In addition, some articles summarized the significant differences between protein corona forming ex vivo and in vivo and emphasized the importance of correct experimental conditions in protein corona research [119,120].

Another challenge is precisely establishing and characterizing the designed NP system in vitro and in vivo [121]. The complexity of the fabrication process and preparation methods of NP systems with biomimetic approaches presents a serious challenge to researchers because the NP systems can play the expected therapeutic role we hope only if they can be constructed precisely. The stability, reproducibility, and homogeneity of the biomimetic NP system also pose a challenge to clinical translation [122]. In the laboratory research and development stage, the product preparation process can be optimized and repeated easily. However, during the scale-up phase, given the sophisticated productive process of NPs, ensuring consistency of quality from batch to batch and developing a protocol for assessing repeatability between batches quickly and accurately becomes challenging [123,124]. These inconsistency issues will influence the result of the clinical study, contribute to potential biases, and ultimately impact the following stage of optimization and clinical translation. It will be the future trend to achieve the same effect from laboratory to clinic and realize the large-scale commercialization of nanomedicine products, and a promising method is simplifying the NP systems [125,126] and developing robust and versatile fabrication approaches [127].

On the one hand, the NP systems with simplified design and components will not only reduce the demand for production technology and equipment but also reduce the clinical research workload for accessing the biocompatibility of several components and reduce the cost of production because of the fewer components [128]. In addition, the progress of advanced synthetic techniques using high-precision fabrication, such as microfluidic technology and 3D printing, will help the large-scale commercialization of nanomedicine products realize inexpensive and standardized development, showing promise for the reproducibility of nanomedicine studies and application [124]. Considering the subsequent large-scale preparation in the early stage of designing NP systems may also endow nanomedicine with better practical significance and application.

As for the characterization methods in nanomedicine, even though they have been well developed in the past, most of them still took a circuitous route to help us fully understand the NP systems we make, which hinders us from designing and developing new NP systems [129,130]. NP systems that function through the protein corona will also pose an external requirement to characterization methods since they must function under the presence of protein corona, and therefore it is necessary to provide comprehensive NP properties after the formation of the protein corona. In addition, based on the intended utility and the dynamic feature of the protein corona, continued characterization may be crucial and necessary for comprehensively understanding the therapy results of NP systems [130]. General standardization for comparing NP systems with different materials, fabrication processes, and other significant conditions is also an obstructive factor in the development of NP systems [131].

In summary, the protein corona can largely alter the biological fate of NPs, including circulation time, biodistribution, and toxicity. In turn, harnessing protein corona with biomimetic approaches is a promising method for designing novel nanomedicine. By precisely controlling the formation and composition of the protein corona, the NP system can achieve many intriguing properties, such as a lower uptake rate into MPS organs, longer circulation time, and targeting ability. Until now, lots of researchers have achieved excellent therapeutic effects with this strategy, but there are still thousands of problems that should be considered and solved before fully understanding and taking advantage of the protein corona, like the mechanism of the protein–NP interaction and protein corona formation under different conditions, the advanced fabrication, and accurate characterization methods.

## Figures and Tables

**Table 1:** Summary of recent studies of biomimetic approaches in nanomedicine to harness protein corona.

Type	Biomimetic Approach	NPs	Mechanism of the Protein Corona Control	Biological Effects	Ref.
**Cell membrane decoration**	RBC membrane decoration	Fe_3_O_4_@RBC NPs	Prevention of protein corona formation	Prolonged circulation time; CD47/SIRP-α signaling pathway	[66]
	RBC membrane decoration	RBC-IMNs	Prevention of protein corona formation	Enhanced CTC targeting ability	[67]
	RBC membrane decoration	CuxO@EM-K	Prevention of protein corona formation	Prolonged circulation time; Retaining Aβ-targeting ability	[68]
	RBC membrane decoration	PDA/BSA/CaCO_3_	Prevention of protein corona formation	Prolonged circulation time	[69]
	RBC membrane decoration	RBC@MMSNs	Prevention of protein corona formation	Prolonged circulation time	[70]
	RBC membrane decoration	RBC-ENPs	Prevention of protein corona formation	Prolonged circulation time; Excellent diffusion ability	[50]
	RBC membrane decoration	FA-RBC-UCNPs	Prevention of protein corona formation	Retain targeting ability	[71]
	RBC membrane decoration	HA&RBCm-LCNPs	Prevention of protein corona formation	Prolonged circulation time; Enhanced specificity to A549 cells	[72]
	WBC membrane decoration	Leukosomes	Prevention of protein corona formation;Promotion of specific proteins adsorption	Prolonged circulation time	[73]
	WBC membrane decoration	NA-Leuko	Prevention of protein corona formation	Prolonged circulation time; Inflamed vasculature Targeting	[74]
	Platelet membrane decoration	Platelet membrane-cloaked nanoparticles	Prevention of protein corona formation	Prolonged circulation time; Enhanced binding to platelet-adhering pathogens	[75]
	Exosomes-based decoration	Hybrid c(RGDm7)-LS-GE/DOX	Prevention of protein corona formation	Prolonged circulation time; CD47/SIRP-α signaling pathway	[76]
	Exosomes-based decoration	DTX@Ang-EM	Prevention of protein corona formation	Prolonged circulation time	[77]
**Endogenous protein coating**	An artificial corona made of human plasma coating	liposomes	Prevention of protein corona formation	Prolonged circulation time; Reduced capture by circulating leukocytes	[78]
	Recombinant fusion protein coating	PCSNs	Prevention of protein corona formation	Prolonged circulation time; Retaining targeting specificity	[54]
	cRGD modified BSA coating	TsR NPs	Prevention of protein corona formation	Enhanced targeting ability to cancer	[79]
	ApoE coating	Graphene	Maintaining a protein corona rich in dysopsonins	Prolonged circulation time; Enhanced enrichment in tumor tissue	[80]
	HSA coating	DRI-S@HSA	Prevention of protein corona formation	Prolonged circulation time; Specific tumor targeting; Deep tumor penetration	[81]
	Surface-bound factor H or SA coating	Graphene-based nanomaterials	Prevention of protein corona formation	Stealth effect	[82]
	SA coating	NR@SA, GTA	Prevention of protein corona formation	Reduction of macrophage phagocytosis; Increasing the interaction with tumor cells	[83]
	Clusterin coating	PS-NPs, HES-NCs	Reducing the IgG absorption	Reduction of the cellar uptake	[84]
	γ-globulins coating	Silica NPs	Promoting a protein corona enriched with opsonins	Impeding the opsonins to their target receptors	[85]
	Folic acid-modified BSA coating	AuNR@EGFP–BSA_FA_, AuNR@RNaseA–BSA_FA_	Prevention of protein corona formation	Prolonged circulation time	[86]
	hydrophobin HFBII	Polystyrene NPs	Prevention of protein corona formation	Reduced aggregation	[87]
	HSA coating	HSA-PIMBs	Prevention of protein corona formation	Excellent enrichment of CTC	[88]
**Biomolecules modification**	Short nontoxic peptide (SP) modification	SP-sLip	Maintaining a protein corona rich in apolipoproteins A1, E, and J	Brain-targeted delivery	[55]
	Peptidomimetic D8 modification	Liposomes	Attenuating the natural IgM absorption	Improved immune compatibility	[89]
	Hyaluronic acid modification	HA-CS NPs	Prevention of protein corona formation	Reduced immunogenicity	[90]
	Retinol modification	RcP NPs	Recruiting the retinol binding protein 4 (RBP) in protein corona	Target delivery to hepatic stellate cells (HSC)	[91]
	Aβ-CN peptide modification	PTX/Aβ-CN-PMs	Forming the ApoE-enriched protein corona	Brain-targeted delivery	[92]
	Phosphorylcholine modification	IONPs	Prevention of protein corona formation	Stealth effect	[93]
	Dihydroartemisinin modification	DHA-NPs	Forming the ApoE-enriched protein corona	Facilitating the tumor accumulation	[94]
	Trivalent cholesterol modification	Chol_3_-Td	Forming the lipoprotein-associated protein corona	Liver target delivery	[95]
	Brushed phosphorylcholine modification	bPC-grafted IONPs	Prevention of protein corona formation	Stealth effect	[96]
	Starch modification	SCS NPs	Prevention of protein corona formation	Prolonged circulation time;	[97]
	Lipid modification	GM3-AVN	Prevention of protein corona formation	Prolonged circulation time; Retaining targeting specificity	[98]
